# Void Suppression Method of CFRP Variable-Thickness Structure Components by Vibration-Assisted Curing Process

**DOI:** 10.3390/polym18101170

**Published:** 2026-05-09

**Authors:** Shunming Yao, Lihua Zhan, Chenglong Guan, Dechao Zhang, Miaomiao Zhang

**Affiliations:** 1 College of Mechanical and Electrical Engineering, Central South University, Changsha 410083, China; yaoshunming@csu.edu.cn; 2 Light Alloys Research Institute, Central South University, Changsha 410083, China; zhangdechao@csu.edu.cn (D.Z.); zhangmmiao11@163.com (M.Z.); 3 School of Mechanical Engineering and Automation, Fuzhou University, Fuzhou 350108, China

**Keywords:** variable-thickness structure, CFRP, vibration-assisted curing process, voids suppression, out-of-autoclave

## Abstract

Composite components with variable-thickness structures often suffer from insufficient forming pressure during curing due to complex pressure transfer in regions with abrupt thickness changes, which easily causes void defects and degrades component performance. In this study, a mechanical vibration-assisted double vacuum bag process is proposed. Finite element analysis of the vibration energy field in saturated porous composites is conducted, and curing experiments for variable-thickness specimens are designed. The effects of vibration, vacuum, and their synergy on void characteristics and mechanical properties are studied using microscopic characterization and mechanical tests. The results indicate that vibration can effectively facilitate gas discharge and accelerate resin flow, while the double vacuum bag process reduces gas discharge resistance in the early curing stage by delaying the vacuum negative pressure application, yet it also results in insufficient resin flow due to this delay. Through the synergistic optimization of vibration-assisted energy field parameters and the double vacuum bag process, gas-induced and flow-induced voids can be effectively suppressed while ensuring curing efficiency, reducing the macroscopic porosity of variable-thickness regions from 8.34% (single vacuum bag process) to 0.43%. This study provides a new approach for the high-quality curing and manufacturing of variable-thickness composite components.

## 1. Introduction

Carbon fiber reinforced polymer (CFRP) composites have become core structural materials in high-end equipment fields such as aerospace, rail transit, and new energy equipment, owing to their outstanding properties including high specific strength, high specific modulus, excellent designability, corrosion resistance, and fatigue resistance [1,2]. To efficiently achieve the synergistic optimization of component lightweighting and load-bearing performance, variable-thickness structural designs have been widely adopted in key load-bearing components such as wing panel skins, spacecraft tank walls, and wind turbine blades. This design enables precise material placement according to load distribution, significantly improving structural efficiency and service reliability [3]. However, variable-thickness components feature large geometric fluctuations and complex ply transitions. During the curing process, the outer fibers tend to bear the majority of the forming pressure, leading to a pressure-unreachable phenomenon in the variable-thickness region. This further induces typical defects such as pores, delamination, resin-rich areas, and resin-starved areas [4,5,6]. Especially in the interlaminar transition region, the abrupt change in fiber orientation and disordered resin flow paths make it easier to form stress concentration and void aggregation [7], which severely restricts the mechanical properties and structural integrity of the components [8].

Numerous studies have confirmed that voids are one of the most critical defects affecting the performance of composite materials, which can significantly reduce interlaminar shear strength [9,10,11], tensile/compressive strength [12,13,14], flexural properties [15,16], and impact toughness [17]. In complex alternating hygrothermal environments, these voids can further accelerate performance degradation, directly threatening the service safety of equipment. Therefore, achieving low-void and high-performance curing of variable-thickness CFRP components is a key technical bottleneck to promote their large-scale application in high-end equipment.

Autoclave curing is currently the mainstream process for manufacturing high-performance composites, as it can provide stable high pressure and a uniform temperature field to effectively suppress void defects. However, with the development of components towards large-scale and integrated manufacturing, the autoclave process is limited by factors such as equipment size, high energy consumption, long cycle time, and high cost, making it difficult to meet the demand for efficient and low-cost manufacturing. In this context, out-of-autoclave (OOA) technology has emerged rapidly. With the advantages of no requirement for autoclaves, flexible manufacturing, low energy consumption, and high efficiency, it has become an important development direction for the manufacturing of large composite components [18,19]. However, OOA curing lacks the uniform and stable pressure field of autoclave processing, resulting in insufficient compaction, inadequate resin flow, difficult gas evacuation, and uncontrolled void growth, which further leads to high component void ratio and weak interfacial bonding. Especially in complex structural regions such as variable-thickness areas, the difficulty of defect control is significantly increased, making it hard to directly meet the requirements of high-performance components [20,21,22,23,24].

To break through the defect control challenge in OOA curing, scholars have carried out extensive research on directions such as prepreg system optimization [25], vacuum-assisted process improvement, and special energy field-assisted curing [26,27]. Among these, vibration-assisted curing introduces a mechanical vibration energy field, which reduces resin viscosity, enhances resin fluidity, accelerates bubble escape, and improves fiber wetting. It has a significant effect on suppressing voids and improving densification under low-pressure forming conditions [28,29,30]. Existing research on vibration-assisted forming mainly focuses on two directions: ultrasonic vibration and mechanical vibration. Ultrasonic vibration is mostly used in liquid-forming processes such as resin transfer molding (RTM), using the cavitation effect to enhance exhaust and wetting [31,32]. Low-frequency mechanical vibration is more suitable for prepreg OOA curing systems, as it can continuously improve flow and exhaust behavior during the resin viscosity change interval, and has been proven to control component void ratio at a low level [27,28,29,30,33].

However, most existing studies analyze the influence of vibration on forming quality based on macroscopic experimental phenomena, lacking the revelation of the vibration action mechanism. A few mechanistic studies only consider vibration propagation in pure resin, ignoring the influences of time-varying viscosity of resin during curing and the saturated porous medium structure formed by fiber and resin on vibration attenuation, energy field distribution, and energy transfer, making it difficult to accurately describe the distribution law of vibration inside variable-thickness components. Meanwhile, existing vibration-assisted processes are mostly applied to material pretreatment before curing, and no composite curing process based on low-frequency mechanical vibration has been reported.

Therefore, aiming at the difficulty of void control in out-of-autoclave (OOA) curing of variable-thickness CFRP components and the technical limitations of vibration-assisted and OOA curing processes, this paper proposes a novel synergistic method of vibration curing and double vacuum bag. Through finite element simulation of the vibration energy field based on the propagation characteristics of composite saturated porous media, the distribution characteristics of the vibration energy field in variable-thickness components are analyzed. Meanwhile, a multi-process comparative experiment of variable-thickness components is designed. Combined with metallographic characterization and interlaminar shear performance testing, the effects of vibration, double vacuum bag, and their synergistic effect on void evolution, resin flow, and mechanical properties are systematically revealed, and the intrinsic mechanism of void suppression in variable-thickness components by the synergistic process is clarified. Finally, a low-defect and high-performance curing process of variable-thickness CFRP components is realized, providing theoretical basis and technical support for high-quality out-of-autoclave manufacturing of variable-thickness composite structures.

## 2. Materials and Methods

### 2.1. Manufacturing Method of Variable-Thickness Components

#### 2.1.1. Materials

This study employed T800 carbon fiber/TRE231 epoxy matrix composite prepreg as the experimental material. The T800/TRE231 prepreg, with a fiber volume fraction of 58% and a resin volume fraction of 42%, was supplied by Jiangsu Changzhou Tianqi Xinxin Technology Co., Ltd., Changzhou, China. The areal density of this prepreg is 130 g/m^2^, and the initial thickness of a single prepreg ply is 0.125 mm.

**Figure 1:**
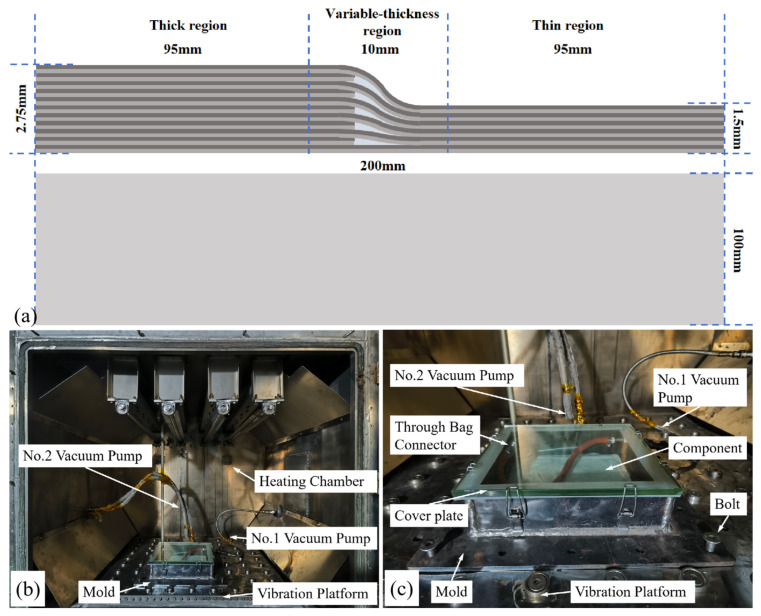
Experimental preparation: (**a**) schematic of layup and dimensions for the variable-thickness component; (**b**) overall view of the integrated heating–vibration experimental platform; (**c**) mold mounted on the vibration table.

In this study, variable-thickness composite components were investigated. The variable-thickness components were fabricated using interlayer layup, as shown in Figure 1a. The thick region consisted of 22 orthogonal plies with a stacking sequence of [0/90]_11s_, with dimensions of 100 × 100 mm and a total thickness of 2.75 mm. The thin region consisted of 12 orthogonal plies with a stacking sequence of [0/90]_6s_, with dimensions of 100 × 100 mm and a total thickness of 1.5 mm. The overall dimension of the variable-thickness component was 200 × 100 mm. The variable-thickness region was defined as the central 10 × 100 mm area of the component, to facilitate void ratio statistics and analyses of geometric dimension changes.

#### 2.1.2. Experimental Platform

Four curing forming processes were adopted in this study: the conventional vacuum bag only (VBO) process, the double vacuum bag (DVB) process, the vibration curing (VC) process with single vacuum bag sealed, and the DVBVC synergistic process combining double vacuum bag and vibration. A self-developed integrated heating–vibration experimental platform was used in the experiments. The heating chamber provided the curing temperature, and a pneumatic vibration table installed below the chamber provided the vibration energy field required for curing forming. The pneumatic vibration table can provide broadband mechanical vibration with a frequency range of 10~5000 Hz. Two independent vacuum systems were set in the heating chamber to meet the requirements of the double vacuum bag process. The specially designed mold for the double vacuum bag process was made of 45# steel and was mounted on the pneumatic vibration table via a bolt connection, to realize the synergistic effect of the double vacuum bag process and the vibration process. The structure of the experimental platform and the mold are shown in Figure 1b,c.

#### 2.1.3. Experimental Methods

**Figure 2:**
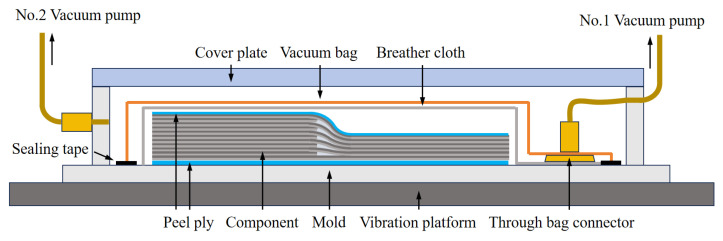
Schematic of the double vacuum bag vibration curing process.

The curing forming process for the variable-thickness composite components was as follows: first, the composite prepreg was laid up on the mold pre-covered with a peel ply according to the layup design. Then, according to the requirements of the VBO curing process, the peel ply, breather cloth, and vacuum bag were laid sequentially for sealing. The vacuum nozzle inside the mold cavity was installed to complete the sealing of the first vacuum bag. After ensuring good sealing inside the first vacuum bag, the transparent glass cover plate was installed on the top of the mold cavity, and the latches were locked to complete the sealing of the second vacuum chamber. After confirming the effectiveness of the two-layer vacuum sealing, the entire mold was installed on the vibration table and mounted with bolt connections. The vacuum system of the first vacuum bag and the vacuum system of the second vacuum chamber were connected separately, completing the pre-curing preparation. The schematic of related process is shown in Figure 2.

**Figure 3:**
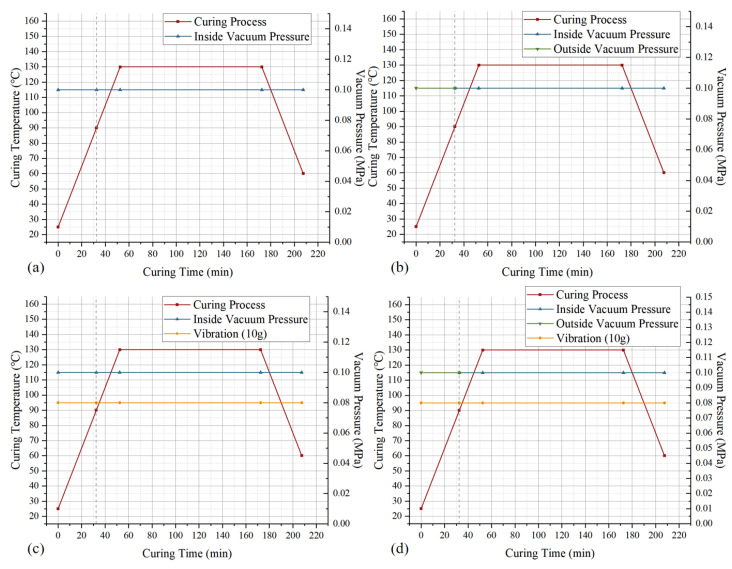
Curing cycles of the processes: (**a**) vacuum bag only (VBO); (**b**) double vacuum bag (DVB); (**c**) vibration curing (VC); (**d**) double vacuum bag vibration curing (DVBVC).

After the above preparation, curing experiments were carried out according to the curing cycles of the double vacuum bag curing process, the vibration curing process, and the double vacuum bag vibration curing process, respectively. Meanwhile, the standard VBO curing process was added as the control. The curing cycles are shown in Figure 3. The heating chamber inside the pneumatic vibration table was heated and held according to the curing process requirements and then cooled to room temperature with air after the holding stage. In addition, for the double vacuum bag process, the vacuum system of the second vacuum chamber turned off the vacuum pump at the process-specified node and opened the vacuum pipeline to let air fully enter, ensuring that the atmospheric pressure outside the first vacuum bag generated sufficient vacuum negative pressure.

### 2.2. Characterization

#### 2.2.1. Metallographic Characterization

**Figure 4:**
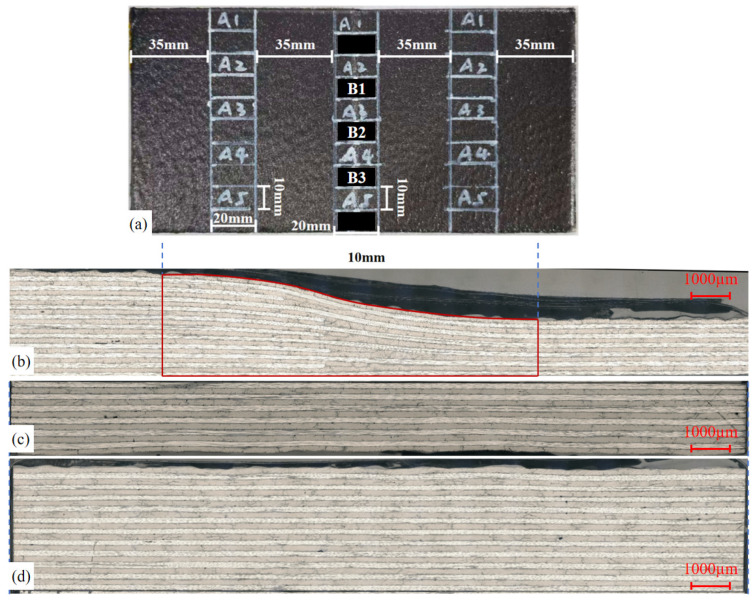
Microscopic observation: (**a**) sampling; (**b**) variable-thickness region; (**c**) thin region; (**d**) thick region.

To observe the changes in voids and geometric dimensions of the variable-thickness components under different processes, an optical digital microscope (ODM, model VHX-5000, KEYENCE, Osaka, Japan) was used in this study. According to the standard GB/T 3365-2008 [34], the formed samples obtained from different curing processes were sampled at the positions shown in Figure 4. For each sample, 5 specimens were taken from the thick region, thin region, and variable-thickness region, respectively. Subsequently, these specimens were polished using waterproof abrasive papers with grit sizes of 1000, 1200, and 1500, and metallographic abrasive papers with grit sizes of 1000, 1600, and 2000. In addition, cross-sections were prepared using a polishing cloth for observation with the high-depth-of-field microscope. The microscopic morphology and size distribution of the cross-sections of the specimens treated with different curing processes were carefully examined and observed. To intuitively compare the difference in void distribution in variable-thickness components under different process conditions, the void ratio of the thick region, thin region, and variable-thickness region of the samples were statistically calculated by ImageJ 1.54P, respectively. To determine the void ratio of the different curing processes and regions, Equation (1) was used:

(1) $$\gamma =\frac{S_{\gamma }}{S_{a}}$$

where γ denotes the void ratio; $S_{\gamma }$ is the area occupied by voids on the cross-section; $S_{a}$ is the cross-sectional area of the specimen.

In addition, to reflect the resin flow during the curing process under different processes, the geometric dimensions of the variable-thickness region after curing were statistically analyzed using the above micrographs. For the variable-thickness region, the outer contour dimensions of the corresponding region were extracted, and the projected area was calculated, as shown in Figure 4. Among them, the A-series samples were the samples for microscopic observation, and the B-series samples were the samples for mechanical property testing. All sample data were statistically analyzed to calculate the average geometric dimensions.

#### 2.2.2. Mechanical Property Testing

Although there is no general mechanical property testing standard for variable-thickness components, this study still referred to the laminates testing method to conduct shear performance testing on the variable-thickness region of the components. This allowed horizontal comparison of the performance changes in variable-thickness components under different curing processes, and comprehensive analysis of the influence of different curing processes on the forming quality of composite materials.

**Figure 5:**
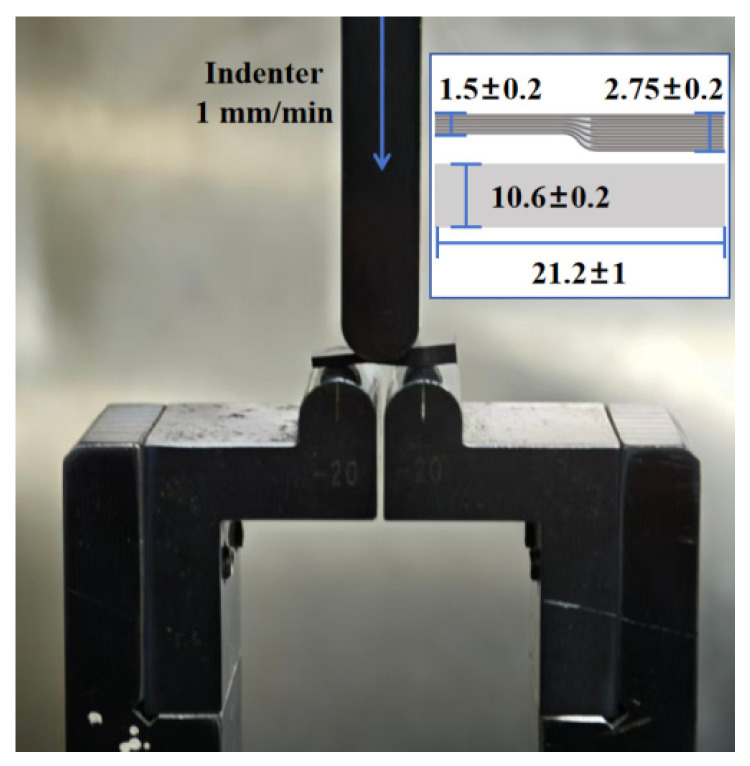
The short-beam three-point bending testing.

To improve data accuracy, 3 specimens were selected for testing for each curing process, and the specimen dimensions are shown in Figure 5. The shear strength testing process referred to the Chinese industry standard JC/T 773—2010 [35] for shear testing of laminates. The interlaminar shear strength was calculated using Equation (2):

(2) $$\tau_{m}=\frac{2F_{max}}{b\left(H+h\right)}$$

where $\tau_{m}$ is the interlaminar shear strength; $F_{max}$ is the maximum load; *b* is the width of the specimen, and *H*, *h* are the thick region and thin region thickness of the specimen, respectively.

Figure 5 shows the short-beam three-point bending testing process for the variable-thickness specimens. ACMT5105 electronic universal testing machine (SANS Testing Machine Co., Ltd., Zhuhai, Guangdong, China) with a force range of 5~10 t was used as the experimental equipment. After the indenter initially contacted the surface of the composite specimen, the indenter pressed down at a rate of 1 mm/min until delamination failure occurred in the composite. The lower horizontal surface of the variable-thickness specimen faces upward to contact the indenter. Meanwhile, a vernier caliper with an accuracy of 0.1 mm is used to position the specimen, ensuring the central axis of the indenter is aligned with the center of the specimen.

## 3. Finite Element Analysis

In this study, combined with Biot’s theory of saturated porous media and the orthotropic constitutive relation of composite materials [36,37,38], the wave equation for orthotropic composite saturated porous media was established:

(3) $$\left\{\begin{array}{c} \rho \frac{\partial^{2}u_{x_{1}}}{\partial t^{2}}+\rho_{f}\frac{\partial^{2}W_{x_{1}}}{\partial t^{2}}=A_{11}\frac{\partial^{2}u_{x_{1}}}{\partial x_{1}^{2}}+A_{12}\frac{\partial^{2}u_{x_{2}}}{\partial x_{1}\partial x_{2}}+A_{13}\frac{\partial^{2}u_{x_{3}}}{\partial x_{1}\partial x_{3}}+\frac{M_{1}}{M}\left(\frac{\partial p_{w}}{\partial x_{1}}-M_{1}\frac{\partial^{2}u_{x_{1}}}{\partial x_{1}^{2}}-M_{2}\frac{\partial^{2}u_{x_{2}}}{\partial x_{1}\partial x_{2}}-M_{3}\frac{\partial^{2}u_{x_{3}}}{\partial x_{1}\partial x_{3}}\right)\\ \;\;\;\;\;\;\;\;\;+2A_{66}\left(\frac{\partial^{2}u_{x_{2}}}{\partial x_{1}\partial x_{2}}+\frac{\partial^{2}u_{x_{1}}}{\partial x_{2}^{2}}\right)+2A_{55}\left(\frac{\partial^{2}u_{x_{3}}}{\partial x_{1}\partial x_{3}}+\frac{\partial^{2}u_{x_{1}}}{\partial x_{3}^{2}}\right)\\ \rho \frac{\partial^{2}u_{x_{2}}}{\partial t^{2}}+\rho_{f}\frac{\partial^{2}W_{x_{2}}}{\partial t^{2}}=A_{12}\frac{\partial^{2}u_{x_{1}}}{\partial x_{1}\partial x_{2}}+A_{22}\frac{\partial^{2}u_{x_{2}}}{\partial x_{2}^{2}}+A_{23}\frac{\partial^{2}u_{x_{3}}}{\partial x_{2}\partial x_{3}}+\frac{M_{2}}{M}\left(\frac{\partial p_{w}}{\partial x_{2}}-M_{1}\frac{\partial^{2}u_{x_{1}}}{\partial x_{1}\partial x_{2}}-M_{2}\frac{\partial^{2}u_{x_{2}}}{\partial x_{2}^{2}}-M_{3}\frac{\partial^{2}u_{x_{3}}}{\partial x_{2}\partial x_{3}}\right)\\ \;\;\;\;\;\;\;\;\;+2A_{66}\left(\frac{\partial^{2}u_{x_{2}}}{\partial x_{1}^{2}}+\frac{\partial^{2}u_{x_{1}}}{\partial x_{1}\partial x_{2}}\right)+2A_{44}\left(\frac{\partial^{2}u_{x_{2}}}{\partial x_{2}\partial x_{3}}+\frac{\partial^{2}u_{x_{3}}}{\partial x_{3}^{2}}\right)\\ \rho \frac{\partial^{2}u_{x_{3}}}{\partial t^{2}}+\rho_{f}\frac{\partial^{2}W_{x_{3}}}{\partial t^{2}}=A_{13}\frac{\partial^{2}u_{x_{1}}}{\partial x_{1}\partial x_{3}}+A_{23}\frac{\partial^{2}u_{x_{2}}}{\partial x_{2}\partial x_{3}}+A_{33}\frac{\partial^{2}u_{x_{3}}}{\partial x_{3}^{2}}+\frac{M_{3}}{M}\left(\frac{\partial p_{w}}{\partial x_{3}}-M_{1}\frac{\partial^{2}u_{x_{1}}}{\partial x_{1}\partial x_{3}}-M_{2}\frac{\partial^{2}u_{x_{2}}}{\partial x_{2}\partial x_{3}}-M_{3}\frac{\partial^{2}u_{x_{3}}}{\partial x_{3}^{2}}\right)\\ \;\;\;\;\;\;\;\;\;+2A_{55}\left(\frac{\partial^{2}u_{x_{3}}}{\partial {x_{1}}^{2}}+\frac{\partial^{2}u_{x_{1}}}{\partial x_{1}\partial x_{3}}\right)+2A_{44}\left(\frac{\partial^{2}u_{x_{2}}}{\partial x_{2}\partial x_{3}}+\frac{\partial^{2}u_{x_{3}}}{\partial x_{2}^{2}}\right)\\ \;\;\;\;\;\;-\frac{\partial p_{w}}{\partial x_{1}}=\rho_{f}\frac{\partial^{2}u_{x_{1}}}{\partial t^{2}}+m_{1}\frac{\partial^{2}W_{x_{1}}}{\partial t^{2}}+r_{1}\frac{\partial W_{x_{1}}}{\partial t}\\ \;\;\;\;\;\;-\frac{\partial p_{w}}{\partial x_{2}}=\rho_{f}\frac{\partial^{2}u_{x_{2}}}{\partial t^{2}}+m_{2}\frac{\partial^{2}W_{x_{2}}}{\partial t^{2}}+r_{2}\frac{\partial W_{x_{2}}}{\partial t}\\ \;\;\;\;\;\;-\frac{\partial p_{w}}{\partial x_{3}}=\rho_{f}\frac{\partial^{2}u_{x_{3}}}{\partial t^{2}}+m_{3}\frac{\partial^{2}W_{x_{3}}}{\partial t^{2}}+r_{3}\frac{\partial W_{x_{3}}}{\partial t} \end{array}\right.$$

where $\sigma_{x_{i}}$ and $\tau_{x_{i}x_{j}}$ are the stress components of the solid skeleton; $p_{w}$ is the pore pressure of the saturated fluid; $A_{11}$, $A_{12}$, $A_{13}$, $A_{22}$, $A_{23}$, $A_{33}$, $A_{44}$, $A_{55}$, $A_{66}$, $M_{1}$, $M_{2}$, $M_{3}$, and M correspond to the 13 independent elastic parameters associated with the properties of the solid framework and pore fluid in orthotropic porous media, where $W_{x_{i}}=\phi \left(U_{i}-u_{i}\right)$ is the displacement of the pore fluid relative to the solid framework; ϕ is the porosity; $U_{i}$ is the displacement of the pore fluid; and $u_{i}$ is the displacement of the solid framework; i,j=1,2,3; $\rho =\left(1-\phi \right)\rho_{s}+\phi \rho_{f}$ represents the average density of the two-phase medium; $\rho_{s}$ represents the density of the solid framework; $\rho_{f}$ represents the density of the pore fluid; $m_{j},r_{j}\left(j=1,2,3\right)$ represents the Biot dissipation coefficient. These variables are functions of the wave frequency and can be expressed by Equation (4):

(4) $$\left\{\begin{array}{c} m_{j}=\frac{\rho_{f}}{\phi }\alpha_{j}\left(\omega \right)\\ r_{j}=\frac{\eta_{w}}{K_{j}\left(\omega \right)}\\ \alpha_{j}\left(\omega \right)=\frac{i\eta_{w}\phi }{K_{j}\left(\omega \right)\omega \rho_{f}}\\ K_{j}\left(\omega \right)=\frac{k_{0}}{{\left\{1-\frac{4i\alpha_{\infty }^{2}k_{0}^{2}\rho_{f}\omega }{\eta_{w}\Lambda^{2}\phi^{2}}\right\}}^{\frac{1}{2}}-\frac{i\alpha_{\infty }k_{0}\rho_{f}\omega }{\eta_{w}\phi }} \end{array}\right.$$

where i represents the imaginary unit, $\alpha_{j}\left(\omega \right)$ represents the dynamic pore curvature, $K_{j}\left(\omega \right)$ represents the dynamic permeability, $\eta_{w}$ represents the dynamic viscosity coefficient (dynamic viscosity), $\alpha_{\infty }$ represents the dynamic pore curvature at infinite frequency (equivalent to the tortuosity factor τ discussed below), $k_{0}$ represents the permeability at ω=0, and Λ represents the characteristic pore size.

Based on the above theoretical model, this study used COMSOL Multiphysics 6.2 simulation software to analyze the vibration propagation during the curing process of fiber-reinforced composite preforms. The material parameters required for the analysis are shown in Table 1.

**Figure 6:**
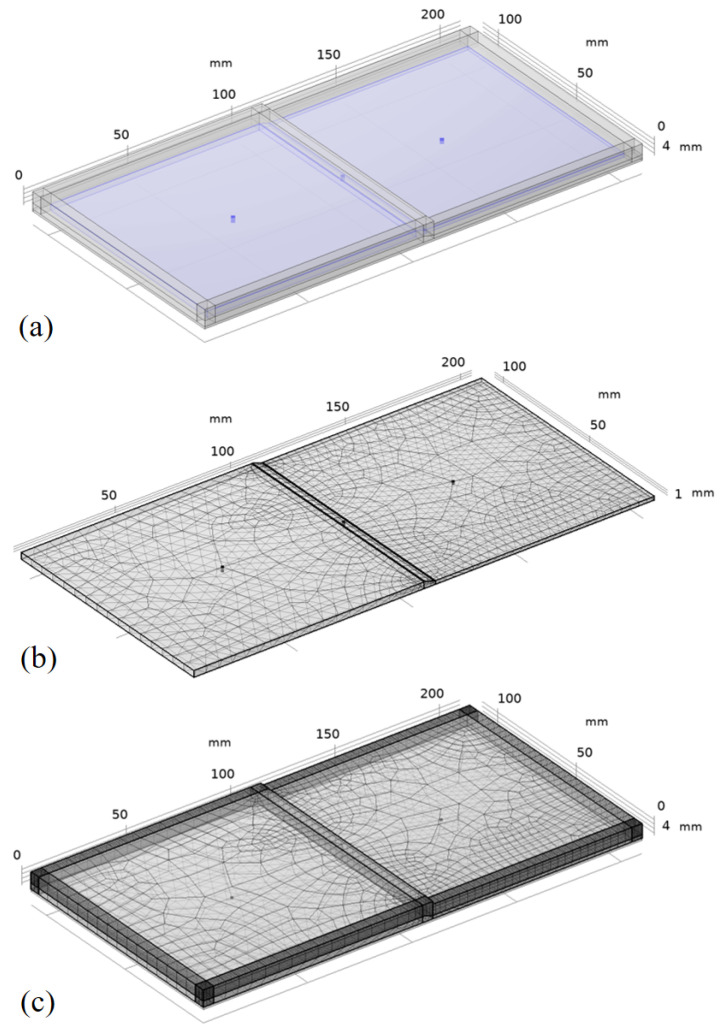
Finite element analysis of the vibration propagation process: (**a**) geometric model of the variable-thickness component; (**b**) mesh division of the component; (**c**) mesh division of the PML.

To accurately reflect the characteristics of the variable-thickness structure, a geometric model of the variable-thickness laminate preform with dimensions of 200 × 100 mm (length × width) and layup referring to the experimental design was established. A 10 mm perfectly matched layer (PML) was set on the outer layer to prevent the echo generated by the hard boundary from affecting the model analysis, as shown in Figure 6a. For meshing, free tetrahedral mesh was used for the component grid elements, as shown in Figure 6b, and automatic swept mesh was used for the PML, as shown in Figure 6c. According to the vibration frequency range of the pneumatic vibration table, the sweep frequency range of the model was set to 1 to 5001 Hz, with a sweep interval of 50 Hz.

To realize the vibration propagation analysis under the process conditions of the vibration table, a zero-phase Ricker wavelet was used as the vibration excitation to investigate the vibration response in the frequency domain. The expression of the Ricker wavelet in the frequency domain is as follows:

(5) $$R(f)=\frac{2f^{2}}{\sqrt{\pi }f_{main}^{3}}e^{-\frac{f^{2}}{f_{main}^{2}}}e^{-j2\pi f}$$

where $f_{0}$ is the dominant frequency of the Ricker wavelet.

**Figure 7:**
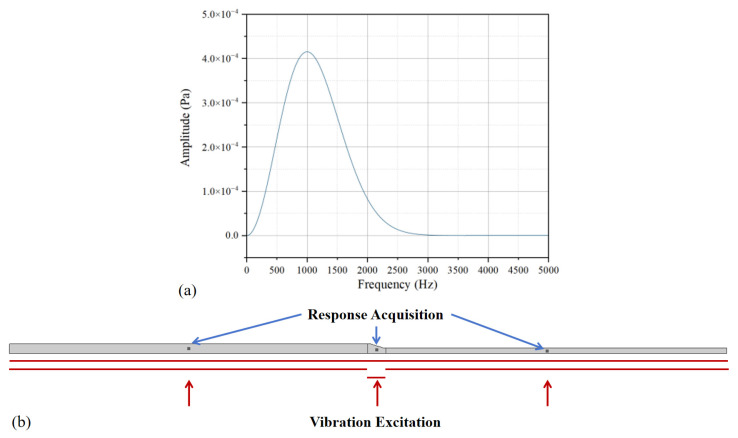
Ricker wavelet analysis: (**a**) spectrum of the 1000 Hz dominant frequency; (**b**) vibration excitation and response acquisition.

Referring to the spectrum range of Ricker wavelets with different dominant frequencies, a Ricker wavelet with a dominant frequency of 1000 Hz was selected as the vibration excitation for analysis. The vibration excitation was applied in three forms: the entire bottom surface, the thick and thin regions of the bottom surface, and the variable-thickness region of the bottom surface. The center points at the half-length position of the thick region, thin region, and variable-thickness region of the component were taken as the monitoring points for response data, to analyze the changes in the response spectrum, as shown in Figure 7.

## 4. Results and Discussion

### 4.1. Influence of Vibration

In OOA processes, due to the lack of a high-pressure forming environment, the forming of components relies on vacuum pressure difference to realize the discharge of gas within and between plies. Then, by increasing the curing temperature, the resin viscosity is reduced, promoting resin flow to wet the emptied channel pores, and finally achieving densification. According to this process characteristic, the void formation mechanisms in OOA processes can be mainly divided into two categories: gas-induced and flow-induced. Gas-induced voids are formed by the residual air trapped inside the composite preform and the volatiles of the resin that cannot be completely discharged, which mostly occur during the pretreatment stages such as layup and the early curing stage when the resin viscosity is high. Flow-induced voids are mainly formed during the process of resin flow wetting the exhaust channels, where uneven resin flow or insufficient wetting leaves cavities after resin gelation. These mostly occur in the middle and later stages when the resin viscosity rises rapidly. Meanwhile, insufficient resin flow and wetting will also affect the interfacial properties of the composite materials.

Mechanical vibration has been proven to have a significant effect on suppressing voids during composite curing. From the perspective of the OOA curing process, for gas-induced voids, mechanical vibration can enhance the dynamic pressure fluctuation of the resin inside the composite, accelerate the gas diffusion process, and promote the escape of trapped gas and volatiles. For flow-induced voids, vibration can improve the uniformity of resin distribution by enhancing resin fluidity, reducing voids caused by local wetting lag. Meanwhile, it accelerates the wetting process of resin on fiber bundles at the mesoscale. Especially in the variable-thickness region, it avoids void formation by improving resin fluidity in the complex structural region.

**Figure 8:**
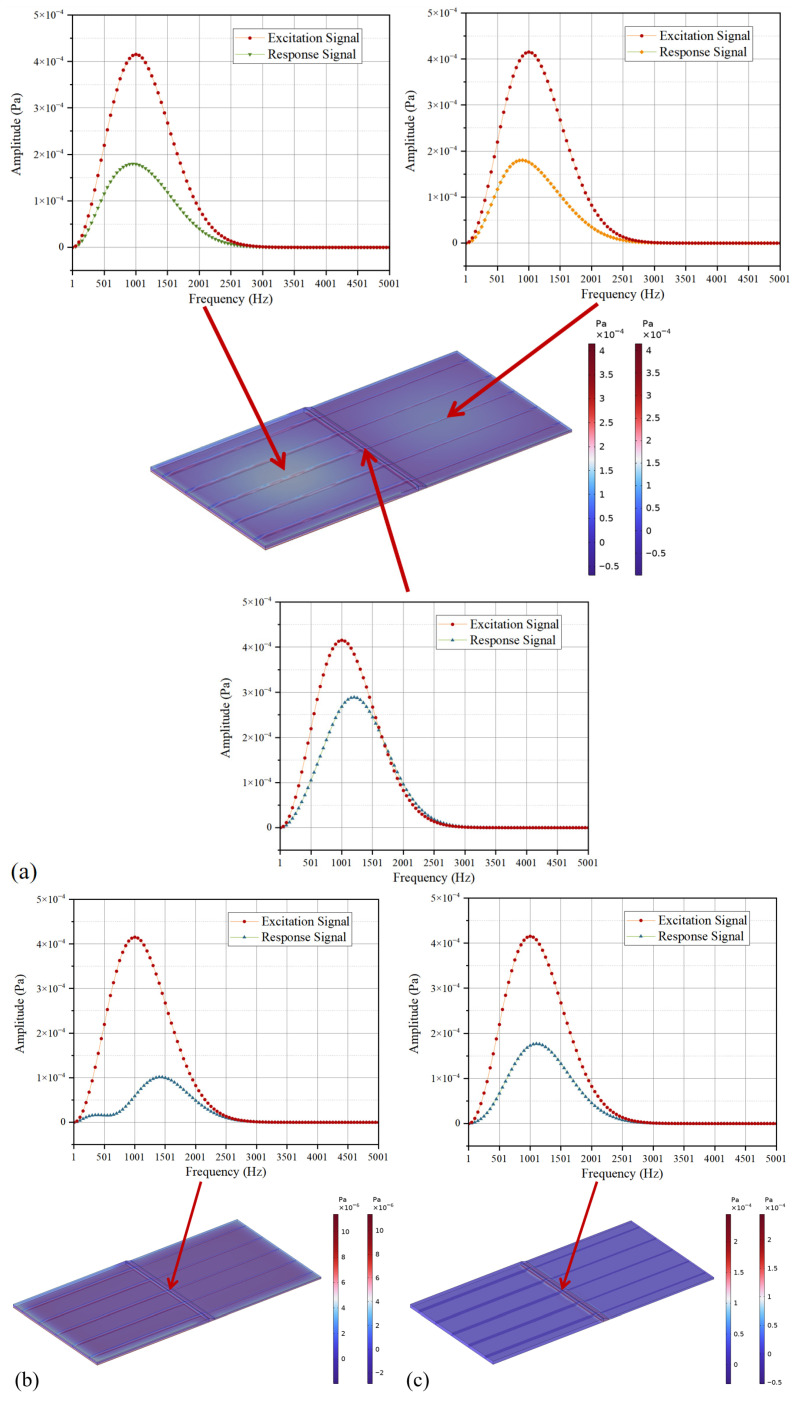
Ricker wavelet analysis: (**a**) entire bottom surface excitation; (**b**) the thick and thin regions of the bottom surface excitation; (**c**) the variable-thickness region of the bottom surface excitation.

Therefore, the distribution of the vibration energy field in the variable-thickness component determines the magnitude of the vibration effect on different regions. Figure 8a shows the sound pressure response of the variable-thickness component under the excitation of the Ricker wavelet on the entire bottom surface. In the middle layer of the component, the sound pressure amplitude of the vibration response in the thick region and thin region is about 40% of the excitation signal amplitude, while that in the variable-thickness region is about 70% of the excitation signal amplitude. Separate analysis of the response sound pressure distribution under the vibration excitation of the thick and thin regions shows that the vibrations of the two regions have a superposition gain in the variable-thickness region, as shown in Figure 8b. Separate analysis of the response sound pressure distribution under the vibration excitation of the variable-thickness region shows that the response in the variable-thickness region is similar to the response in the thick and thin regions under the overall excitation, as shown in Figure 8c. Therefore, the vibration response in the variable-thickness region is subject to a greater effect from the vibration energy field, due to the gain from the thick and thin regions.

**Figure 9:**
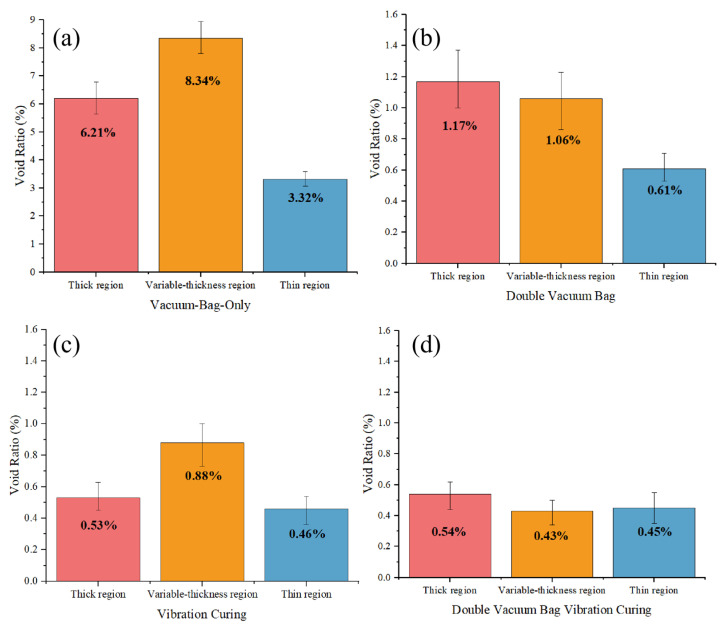
Comparison of void ratios: (**a**) vacuum bag only (VBO); (**b**) double vacuum bag (DVB); (**c**) vibration curing (VC); (**d**) double vacuum bag vibration curing (DVBVC).

Nevertheless, the effect of vibration on the evolution law of voids does not follow a simple linear relationship. Figure 9 compares the void ratio differences in various regions under different processes. As shown in Figure 9a, the void ratio of the variable-thickness region cured via the VBO process is significantly higher than that of the thin and thick regions. This is attributed to the complex interlayer hybrid structure in the variable-thickness region, which leads to disordered gas permeation and resin flow paths. Massive, closed voids are readily formed due to gas retention, and uneven resin impregnation also induces entrapped bubbles.

In contrast, the overall void ratio of component fabricated by the DVB process in Figure 9b is greatly reduced, with void ratio gradually decreasing from the thick region to the variable-thickness region and further to the thin region. This phenomenon can be explained by the inherent characteristics of the DVB process, a specialized technique targeted at gas-induced voids. In the VBO process, the vacuum negative pressure compresses gas escape channels at the initial curing stage, hindering gas discharge. By contrast, the DVB process avoids applying vacuum negative pressure to components before the reduction in resin viscosity, enabling smooth gas escape to the maximum extent. However, the absence of vacuum-driven compaction during the resin viscosity reduction stage results in insufficient resin flow and compaction of the laminate, thereby increasing flow-induced voids. Consequently, its void distribution is highly consistent with the resin flow pattern: the greater the thickness, the weaker the flow compaction effect, and the higher the void ratio.

Figure 9c illustrates the characteristics of the VC process, which simultaneously facilitates gas escape and resin flow. Its overall void ratio is lower than that of the VBO process, yet it exhibits a similar void distribution feature—higher void ratio in the variable-thickness region. Even simulation results confirm that the attenuation of the vibration energy field is lower in the variable-thickness region, this issue cannot be resolved without adjusting the curing cycle.

**Figure 10:**
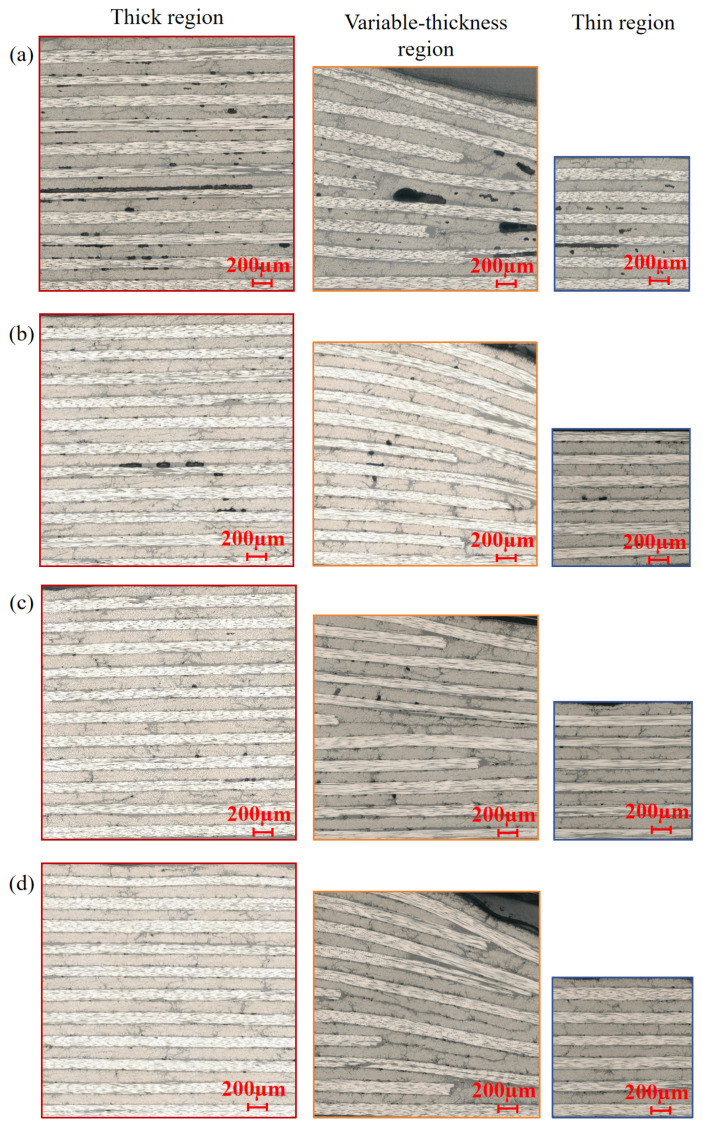
Microscopic morphology in different regions: (**a**) vacuum bag only (VBO); (**b**) double vacuum bag (DVB); (**c**) vibration curing (VC); (**d**) double vacuum bag vibration curing (DVBVC).

The DVBVC process integrates the advantages of both DVB and VC processes. As presented in Figure 9d, compared with the VC process, DVBVC further reduces the void ratio in the variable-thickness region, achieving a relatively uniform void distribution and a low overall void ratio across the entire component. The void distribution characteristics under different processes are further verified by the microscopic images in Figure 10.

Furthermore, comparative analysis of void ratio in different regions in Figure 9 reveals that although the VC process reduces the overall void ratio of components, the void ratio in the variable-thickness region remains relatively high. This indicates that the suppression effect of vibration on voids exhibits a time-dependent cumulative effect. Even a stronger vibration energy field requires a matched action duration to reduce the void ratio of the variable-thickness region to the level of uniform-thickness regions.

Meanwhile, as observed in Figure 9d and Figure 10d, the reduction in void ratio presents a marginal effect. The void ratio remains basically stable after dropping to approximately 0.5%, which meets the strict void ratio requirements for load-bearing composite components in the aerospace industry. Therefore, for variable-thickness components, the synergistic combination of DVB and VC processes can effectively lower the overall void ratio without modifying the curing procedure and extending the curing time to accumulate the vibration modification effect on high void ratio regions.

**Figure 11:**
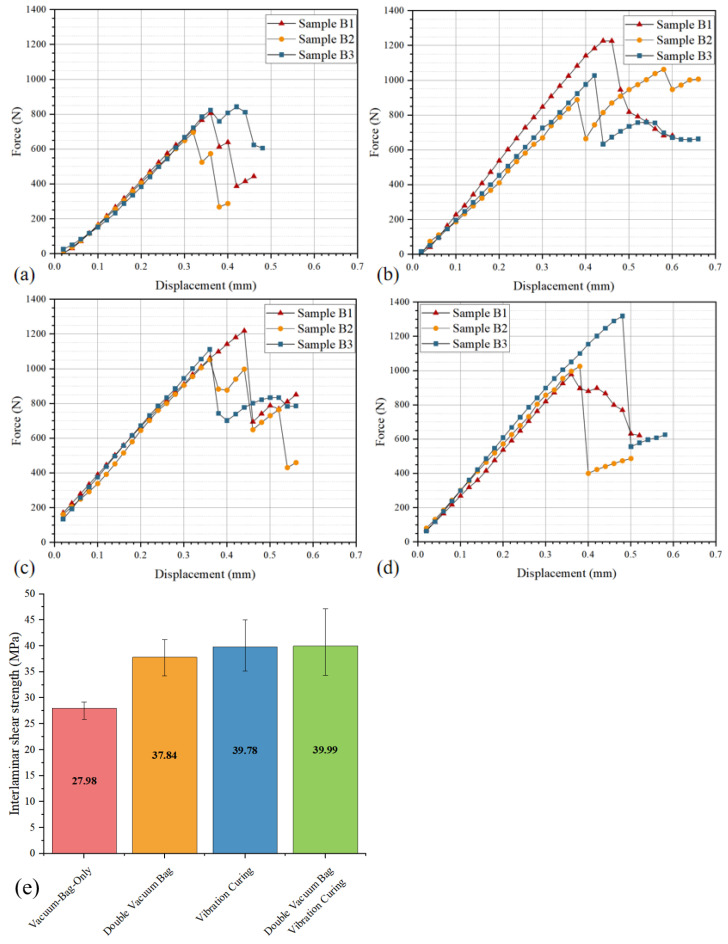
Comparison of interlaminar shear properties: (**a**) force–displacement curve of VBO; (**b**) force–displacement curve of DVB; (**c**) force–displacement curve of VC; (**d**) force–displacement curve of DVBVC; (**e**) results of interlaminar shear strength under different processes.

In addition, the differences in interlaminar shear strength and geometric dimensions between the VBO process and the vibration process also illustrate the positive effect of vibration on the interfacial properties of the variable-thickness region. As shown in Figure 11, the interlaminar shear strength of the VBO process is about 60% of that of the vibration process. This proves that vibration not only effectively suppresses void formation but also promotes the sufficient flow of resin in the variable-thickness region and sufficient wetting with fiber bundles, thereby improving the interfacial properties of the variable-thickness region. Meanwhile, the projected area of the variable-thickness region in the vibration process is smaller, proving that vibration has a positive promoting effect on the resin flow of the entire variable-thickness component.

**Figure 12:**
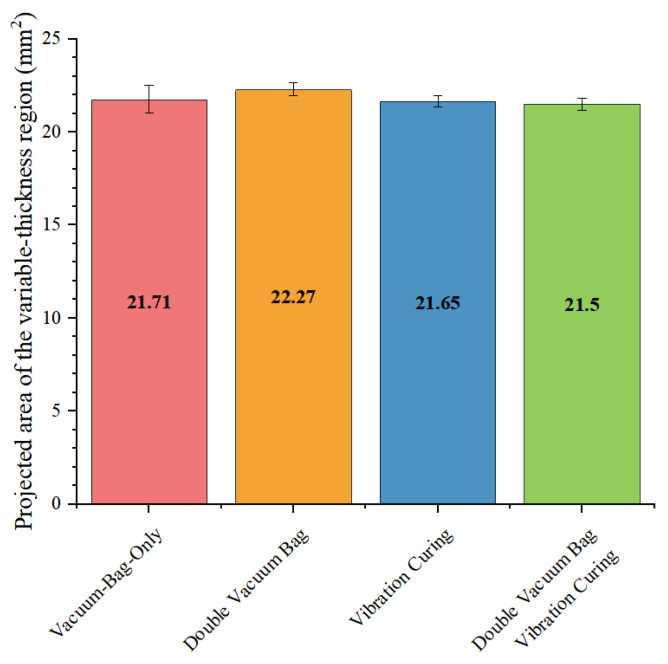
Comparison of projected area.

Similarly, as observed in Figure 12, the difference in the projected area of the variable-thickness region between the vibration process and the VBO process is small, which proves that the influence of vibration on resin flow also has a marginal effect. This may be due to the increase in the fiber volume fraction and the dynamic permeability of the porous medium after the resin gradually flows out.

### 4.2. Influence of Vacuum

In the VBO process, the vacuum environment inside the vacuum bag provides the driving force for gas discharge, and the vacuum bag film applies uniform pressure on the resin to promote its flow and wetting, which corresponds to the formation mechanisms of gas-induced and flow-induced voids, respectively. However, in the VBO process, to apply the vacuum environment, it is necessary to seal the component with a vacuum bag film. During the discharge of gas-induced voids, the pressure from the vacuum bag film does not accelerate the discharge of air. On the contrary, it compresses the preform, leading to the compression or even closure of open pores, inhibiting the ballooning effect of gas-containing voids under negative pressure, thereby hindering the smooth escape of internal gas. Therefore, to avoid the blockage of exhaust channels caused by premature application of vacuum pressure, the VBO process usually adds a pre-treatment vacuum pumping stage before curing heating, to conduct initial vacuum pumping when the resin viscosity has not yet decreased, increasing the time window for gas discharge. However, for variable-thickness components, due to the complex structure of the variable-thickness region, there are large gaps between the fiber layers after layup which are also filled with air. The pre-vacuum process can hardly completely avoid the premature compression of the preform by the vacuum bag pressure. After compression, more gas is trapped in the variable-thickness region, leading to larger residual voids.

To avoid the influence of vacuum bag pressure on gas escape, the double vacuum bag process was proposed, as shown in Figure 2. At the beginning of the process, the vacuum pumps for the inner and outer chambers started simultaneously, so that while the inner chamber formed a vacuum environment, the vacuum bag film did not exert negative pressure on the preform. Then, the temperature was raised to 90 °C, where the resin viscosity decreased to a low value. At this point, the vacuum pump for the outer chamber was turned off and connected to the atmospheric environment, so that the inner vacuum bag generated negative pressure. The temperature was held for 30 min to allow sufficient resin flow. Finally, the curing temperature was raised to 130 °C and held for 2 h to complete the curing forming. The double vacuum bag process effectively decoupled the gas-induced stage and the flow-induced stage, realizing the control of the pressing timing in the variable-thickness region, and ensuring smooth exhaust during the gas-induced stage.

As shown in Figure 9, the void ratio of the DVB process is significantly lower than that of the VBO process. Especially in the variable-thickness region, there is small difference in void ratio compared with the thin and thick regions. This proves that the DVB process can effectively alleviate the problem of exhaust channel collapse caused by the compression of the single-layer film in the traditional VBO process. It not only ensures the smooth escape of gas in the early stage but also applies pressure in time during the flow stage to promote wetting. In particular, it improves the filling consistency of the variable-thickness region, greatly reducing the void ratio.

However, in the DVB process, the action time of vacuum pressure is shorter than the curing cycle, which limits the time window for resin flow and wetting. Meanwhile, it also affects the formation time of the microscopic interface between resin and fibers, thereby weakening the interfacial bonding strength. Comparing the interlaminar shear properties of the vibration curing process and the DVB process, although the DVB process can also significantly reduce void ratio, its interlaminar shear strength is lower than that of the components fabricated by the vibration process with similar void ratio. Meanwhile, the geometric dimensions are larger, indicating insufficient resin flow and problems in interfacial bonding performance in the DVB process. Therefore, to maintain the advantage of the DVB process in gas-induced void control, while compensating for its shortcomings in resin flow regulation and interface formation, it is necessary to introduce a new mechanism that neither interferes with the rapid discharge of air during the gas-induced stage, nor actively regulates the resin wetting behavior.

### 4.3. Synergistic Influence of Vibration and Vacuum

From the characteristics of the vibration-assisted curing process and the double vacuum bag process mentioned above, it can be seen that the vibration curing process can effectively improve the resin fluidity and wettability during the flow-induced stage, while the double vacuum bag process can effectively ensure smooth exhaust during the gas-induced stage, especially avoiding the collapse of gas escape channels caused by single-layer film compression in the variable-thickness region. Combining the vibration-assisted process and the double vacuum bag process can take advantage of both processes: During the gas-induced stage, since the negative pressure generated by the vacuum bag film has not been applied, vibration not only does not interfere with the gas escape channels inside the preform but also promotes the rapid migration of gas along low-resistance paths in the vacuum environment, enhancing the initial exhaust efficiency. After entering the flow-induced stage, the synergistic effect of vibration and the negative pressure applied by the vacuum bag film can compensate for the problems of resin fluidity and wettability in the DVB process, suppress flow-induced voids, and improve interfacial properties at the same time.

As shown in Figure 9, Figure 10 and Figure 11, the void ratio, interlaminar shear properties, and projected area of the double vacuum-vibration synergistic process are presented. The void ratio of the synergistic process is further reduced compared with the DVB process and the vibration process, and the void ratio in the variable-thickness region is basically consistent with that in the thin and thick regions. This indicates that the synergistic effect of vibration and vacuum not only effectively suppresses the formation of both gas-induced and flow-induced voids, but also significantly improves the wetting uniformity of resin in complex structural regions. Comparing the interlaminar shear properties, the interlaminar shear strength of the synergistic process has increased by about 100% compared with the VBO process, and has a certain improvement compared with the vibration process, with significantly improved interlaminar bonding quality.

The above results indicate that the synergistic effect of vibration and vacuum not only effectively suppresses dual gas-induced and flow-induced voids, but also promotes sufficient resin wetting and interfacial densification, realizing the synergistic optimization of low void ratio and high interfacial performance. This provides a new approach for the high-quality forming of complex-structure composite materials.

## 5. Conclusions

In this paper, by combining the vibration-assisted curing process and the double vacuum bag process, the curing void defects in variable-thickness CFRP components were effectively suppressed. Through designing curing process experiments for variable-thickness components, combined with metallographic characterization, mechanical property testing, and finite element analysis of vibration energy fields based on the propagation characteristics of composite saturated porous media, the influence of vibration, vacuum, and their synergistic effect on void defects and mechanical properties of composite materials was studied. The specific conclusions are as follows:

Based on the vibration propagation model of composite porous media, the propagation law of the vibration energy field inside the variable-thickness composite component was simulated and analyzed. Combined with the curing process experiments of variable-thickness composite components, it was proved that, compared with the VBO process, the vibration process has a significant void suppression effect, while improving the resin flow and wetting uniformity in the variable-thickness region, and enhancing the interfacial bonding performance.

Based on the experimental results of double vacuum bag curing, the void evolution characteristics of the variable-thickness structure were analyzed. It proved the negative influence of vacuum negative pressure on gas-induced voids, especially in the variable-thickness region. It verified the advantage of the double vacuum bag process in suppressing gas-induced voids in the variable-thickness region and also identified the limitations of the double vacuum bag process in terms of resin fluidity and wettability.

Based on the characteristics of the vibration process and the double vacuum bag process, a double vacuum bag-vibration synergistic curing process was proposed. The curing process experiment results show that, compared with the vibration process and the double vacuum bag process, the synergistic process further reduces the void ratio and improves the interlaminar shear performance, realizing the improvement of the curing forming quality of variable-thickness components. This provides a new idea for the curing forming process of complex-structure composite components.

## Figures and Tables

**Table 1:** Properties of the composite preform.

**Porous Parameters**	
Permeability, $S_{11}$= $S_{22}$	6.72 × 10^−13^
Permeability, $S_{33}$	1.72 × 10^−14^
Tortuosity factor, $\tau_{11}$= $\tau_{22}$	5.1
Tortuosity factor, $\tau_{33}$	7.97
**Mechanical properties**	
Young’s modulus, $E_{11}=E_{22}$/GPa	100.43
Young’s modulus, $E_{33}$/GPa	9.75
Shear modulus, $G_{12}=G_{13}$/GPa	7.15
Shear modulus, $G_{23}$/GPa	4.55
Poisson’s ratio, $\nu_{12}=\nu_{13}$	0.315
Poisson’s ratio, $\nu_{23}$	0.07
**Fluid properties**	
Viscosity $\mu_{r}$/Pa·s	1
Density/kg·m^−3^	1200

## Data Availability

The original contributions presented in this study are included in the article. Further inquiries can be directed to the corresponding authors.
